# CO-DESIGNING CANCER SCREENING INTERVENTIONS WITH PEOPLE OVER 55 YEARS OLD AT RISK FOR LUNG CANCER

**DOI:** 10.1093/geroni/igz038.3344

**Published:** 2019-11-08

**Authors:** Leah Tuzzio, Lorella Palazzo, Sarah Brush, Kelly Ehrlich, Melissa Anderson, Hongyuan Gao, Karen Wernli

**Affiliations:** Kaiser Permanente Washington Health Research Institute, Seattle, Washington, United States

## Abstract

In 2014, the US Preventive Task Force recommended annual lung cancer screening with low dose CT (LDCT) for adults aged 55 to 80 years old with significant smoking history. Although screening reduces lung cancer mortality, the leading cause of cancer mortality in the US, adherence to screening follow-up remains low. In a human-centered design qualitative study, health services researchers and eight adults over 55 years old from Kaiser Permanente Washington who had recently had an LDCT participated in two co-design sessions. We elicited barriers, facilitators and design principles to develop multilevel interventions that aim to improve adherence to ongoing LDCT. In the initial discussion, participants identified four key areas for improvements to adherence: a) reminders for scheduling and appointments, b) knowledge about tests and follow-up, c) convenience in location and scheduling, and d) financial and non-financial incentives. In a second session, participants referenced patient personas and sketched storyboards, a comic strip-like format showing steps in a journey, to describe different ways to help patients return for LDCTs. Through qualitative analysis, we identified ten elements to consider incorporating in multilevel interventions: versatility (e.g., multiple reminder options), social support (e.g., families, peers), individualization (e.g., tailoring to patient needs), feelings (e.g., fear, relief), knowledge (e.g., harms/benefits, expectations), responsibility (e.g., who is accountable for reminders), continuity (e.g., clear pathway to adherence), consistency (e.g., same messages), cadence (e.g., rhythm of messages), and acknowledgment (e.g., recognition of screening completion). Next steps are to incorporate feedback from clinical stakeholders and develop multilevel interventions for further testing.

